# Origin of current intermediate wheatgrass germplasm being developed for Kernza grain production

**DOI:** 10.21203/rs.3.rs-3399539/v1

**Published:** 2023-10-10

**Authors:** Peggy Wagoner, Jared Crain, Steve Larson, Lee DeHaan

**Affiliations:** Rodale Institute; Kansas State University; USDA-ARS, Utah State University; The Land Institute

**Keywords:** intermediate wheatgrass, domestication, perennial grain, Kernza^®^

## Abstract

Intermediate wheatgrass (IWG, *Thinopyrum intermedium* [Host] Barkworth & D. R. Dewey) has been developed as a perennial grain crop to provide ecosystem services, environmental benefits, and human food. Grain and products derived from IWG varieties improved for food production have been marketed under the registered trademark, Kernza. In the 1980s, a joint breeding effort between the Rodale Institute (RI) and the Big Flats Plant Material Center used IWG plant introductions (PI) from the National Plant Germplasm System (NPGS) and recurrent phenotypic selection to improve populations of IWG with the goal of developing a perennial grain. Initial selections were provided to The Land Institute where they were subsequently improved for grain production, yet the identity of the founder material of improved, food-grade IWG has not been publicly documented. Recently recovered original documents have been used to reconstruct the early breeding program to identify the most likely 20 PIs that form the founders of modern food-grade IWG. Molecular data using genotyping-by-sequencing in current elite breeding material, remnant seed from the initial RI selections, and preserved sample material have provided supporting evidence for the historical records. The genetic origin for food-grade IWG is focused between the Black Sea and Caspian Sea in the Stavropol region of Russia, with smaller contributions likely from collections as distant as Kazakhstan in the east to Turkey in the west. This work connects the flow of germplasm and utility of NPGS PIs to present day IWG grain cultivars being developed in multiple breeding programs around the world.

## Introduction

Inspired by Wes Jackson’s ideas (1980) for a diverse, herbaceous perennial polyculture, the Rodale Research Center (now known as Rodale Institute [RI]) in the early 1980s evaluated approximately 300 different species for their utility in perennial agriculture similar to natural ecosystems. Following evaluation of more than 100 different grass species, intermediate wheatgrass (IWG, *Thinopyrum intermedium* (Host) barkworth & D.R. Dewey) was selected in 1985 as having the highest potential to be developed into a perennial grain crop based on its plant structure, seed production characteristics, perennialism, food use potential (Wagoner 1990a), and the fact that it is related to important Triticeae grain crops (Wagoner 1990b) likely indicating a good nutritional profile and lack of anti-nutritive compounds. Prior to selection for domestication, IWG had a long history within the United States for erosion control, forage production (Asay and Jensen 1996), and utilization as a tertiary gene pool for annual wheat improvement (Li and Wang 2009; Pototskaya et al. 2022).

Since the identification of IWG as a target of domestication, a dedicated and expanding research effort has been conducted to bring the idea of perennial grains to fruition. Research on IWG for perennial grain use has focused on all facets of plant breeding, agronomic practices, and potential consumer utilization. Some of this research has shown the environmental benefits posited by perennial crops such as reduced nitrate leaching (Culman et al. 2013; Jungers et al. 2019; Huddell et al. 2023), net carbon accumulation (de Oliveira et al. 2018), increased soil particulate organic matter (van der Pol et al. 2022), and reduced surface runoff of particulate matter (poultry litter) (Katuwal et al. 2023) compared to annual crops.

Supplementing positive environmental benefits of perennial grain, agronomic research has led to a better understanding of plant development (Duchene et al. 2021), management (Jungers et al. 2017, 2022), and harvesting (Tautges et al. 2023). Finally, many food applications have been evaluated including flour quality and processing methods (Marti et al. 2015, 2016; Zhang et al. 2015; Rahardjo et al. 2018; Banjade et al. 2019) as well as studies looking at malting applications (Marcus and Fox 2023). Grain from IWG that has been improved for grain and food production is marketed under the trade name Kernza to clearly distinguish it from seeds of forage-type IWG. Currently, there are a number of commercial Kernza products available to consumers.

A central tenet to the environmental, agronomic, and end use research has been that plant breeding will play a pivotal role in developing higher yielding varieties. Bolstered by early breeding successes that showed up to 77% increase in grain yield in two cycles of selection (DeHaan et al. 2014) and 21st century molecular methods, plant breeding programs are working to develop better performing varieties with a watershed moment in 2019 with the release of ‘MN-Clearwater’ as the world’s first IWG developed for food consumption (Bajgain et al. 2020b). Kernza refers to the grain that is marketed from varieties that have been developed for human consumption of the grain, such as MN-Clearwater, we use “improved IWG” to signify *Thinopyrum intermedium* germplasm bred for human consumption that could produce Kernza grain. Following the early breeding efforts of RI, there are now breeding programs in Canada, Ukraine, Sweden, and the United States actively breeding for improved IWG. In an effort to speed genetic gain, these programs have actively utilized genomic selection (GS) (Zhang et al. 2016; Crain et al. 2021a) to reduce breeding cycle time. Implementation of GS has required molecular methods like genotyping-by-sequencing (GBS) (Elshire et al. 2011; Poland et al. 2012) that have also provided data needed to dissect genetic architecture through biparental quantitative trait loci (QTL) mapping (Zhang et al. 2017; Larson et al. 2019) and association mapping (Bajgain et al. 2019; Altendorf et al. 2021b, a; Bajgain and Anderson 2021; Crain et al. 2022) Taken together, breeding programs have leveraged these newer tools and methodologies to both enhance the agronomic performance of IWG as well as identify causal variants of traits that both assist in breeding and our scientific understanding of IWG genetics.

The basic evaluation and development of improved IWG since the 1980s was previously reported (Zhang et al. 2016) as well as the development of breeding programs and selection methods in programs established after 2001 (DeHaan et al. 2018; Bajgain et al. 2023). As many of the breeding programs have a combination of pedigree or molecular data along with laboratory records, most improved IWG lineages can be traced back to their programs’ beginning material which subsequently traces back to RI selections. For reference, IWG was first introduced into the United States in 1932 (Musil 1948). It is also known that RI evaluated many of the publicly available plant introduction (PI) accessions from the United States Department of Agriculture (USDA) National Plant Germplasm System (NPGS) gene bank (Wagoner 1990a), so there is nearly a direct connection from improved IWG (Kernza) varieties to PIs. As most of the IWG PI accessions in the gene bank have been characterized phenotypically and genotypically (Crain et al. 2023), and include at least some source documentation, there is an opportunity to fill in the missing link between the geographic origin of the PI founders and improved IWG varieties. Using molecular data, remnant seed and plant material, and recently recovered field and laboratory documents, our objective is to solidify the reported early breeding lineages (Zhang et al. 2016; Bajgain et al. 2023) to the NPGS PI material used to develop improved, food-grade IWG.

## Materials and methods

### Historical Records and Experiments

#### Original Material Evaluation

Over the course of nearly two decades, RI was actively involved in all aspects of perennial grain development including species evaluation and selection, germplasm enhancements, agronomic practices, and product utilization. For completeness, the experimental information will be described below where many of these details have been acquired from principal investigators and sources included in Supplementary File 1.

Beginning in 1983, an accession evaluation nursery (hereafter referred to as RI Herbary) of nearly 300 perennial species was established at RI to evaluate plant accessions for their potential use in perennial herbaceous polyculture. Each accession was grown in a single, one-meter-long row separated from other accessions by mowed grass alleyways. Once IWG was selected as the most promising candidate for domestication in 1985, RI obtained more accessions of IWG from a variety of sources including the USDA NPGS gene bank in Pullman, WA, researchers in the United States, seed companies, and from foreign seed banks. Each evaluation row consisted of ten genets from the same PI accession. As each individual plant can have its own unique genetic makeup due to IWG’s outcrossing nature (Jensen et al. 1990), we use the term genet (Zhang et al. 2016) to refer to individual plants of an IWG accession. Using the terminology of Zhang et al., (2016) a single genet can be cloned into multiple ramets (plants) having the same genetic makeup. By 1987, there were 43 unique IWG accessions in the RI Herbary and each accession was evaluated for plant structure and vigor, as well as seed production characteristics including ease of threshing, synchrony of maturity, and shatter resistance. In addition, 100seed weight, as a yield component trait, was determined for each accession by averaging the weight of 3 randomly selected sets of 100 seeds dried to 12% moisture. From 1988–1993, a larger number of IWG accessions were more intensively evaluated annually as described above with the inclusion of two more yield components including seed set rating (SSR, (Trupp and Slinkard 1965) measuring seed head fertility and seed yield (g) per 10 heads. The SSR trait was calculated as the weight (g) of clean seed from 10 seed heads divided by the weight (g) of the unthreshed seed heads. Evaluations at RI emphasized seed size (as measured by 100seed weight) and seed head fertility (measured by SSR) as these measurements were both highly heritable in IWG and a direct correlation had been established between seed yield and seed head fertility (Slinkard 1965).

#### Polycross-1

In 1988, another 99 IWG accessions consisting of 10 genets each were added to the RI Herbary for a total of 139 unique IWG accessions completing the panel of potential candidates for the first polycross (Polycross-1) breeding cycle. Of the evaluated material, 116 accessions were PIs from the USDA NPGS gene bank in Pullman, WA. Selection of parents from the 139 accessions which were evaluated both in 1988 and 1989 was based on favorable seed yield characteristics including 100seed weight, SSR, and seed yield per 10 heads. This multi-year evaluation included both a drought (1988) and above average precipitation (1989). Twenty accessions which represented favorable phenotypic traits were selected. Segments of each selected evaluation row were dug from the field in November 1989. Each segment was divided into three, placed into pots and vernalized during December 1989. It is likely that multiple genets, rather than three ramets, were dug up in the three potted plants per accession as the rhizomes of individual genets (10 per row) would be intermingled and indistinguishable from each other within these selected accession rows. In January 1990, the pots were placed in the RI greenhouse for intercrossing to produce Syn-0 seeds. Pots were rearranged frequently to ensure that pollination could occur between all accessions. Syn-0 seeds produced by this intercrossing were bulked and then planted into market packs, one seed per cell in June 1990. A total of 360 seedlings (genets) were transplanted in September, 1990 at the USDA Natural Resource Conservation Service (NRCS) Big Flats Plant Materials Center (BFPMC), Big Flats, NY, establishing Selection Nursery-1. The collaboration with BFPMC resulted from both an institutional reorganization of RI and the expertise of BFPMC in selection and varietal development of undomesticated species.

#### Reconstructing Selection of 20 Polycross-1 Parents

Although the identity of the 20 accessions selected in Polycross-1 would have been recorded at the time, this information is no longer available. Based on recent success of improved IWG, including a released variety (Bajgain et al. 2020b), there has been interest in identifying these 20 accessions. To reconstruct the most likely selected accessions, recently recovered field and laboratory records have been reviewed to determine the most likely accessions used in Polycross-1 (Supplementary File 1). These records provide details of yield component traits in 1988 and 1989 and specifically state that 20 accessions with favorable yield characteristics were selected for Polycross-1; however, the exact accessions were not provided. There was evidence of genotype-by-environment interaction as a list of 22 accessions that had high yield in 1988 (drought year) were often not the highest yielding in 1989 (wet year). Additionally, the average values combined across the two years for the three yield components of the 20 selected accessions were given as 100seed weight (0.495 g), SSR (45%), and yield per 10 heads (2.5g). These averages provide a target value in searching the records to identify the most likely combination of selected accessions.

#### Polycross-2

From 1991–1994, Selection Nursery-1 was evaluated jointly by RI and BFPMC for the same traits that were emphasized in initial RI evaluations. The 360 genets grown from Polycross-1 seed were maintained as individual genets with bare ground around each plant. Additionally, seed heads were removed from each plant at maturity to prevent contamination by shattered seed. Of the 360 genets, 11 genets were chosen to be included in Polycross-2. While evaluations of Selection Nursery-1 were occurring, RI was actively continuing evaluations and expanding their IWG germplasm collection. In the fall 1988, another 111 IWG lines were added to the RI Herbary with four unique genets per row for each accession. RI researchers selected these accessions in 1987 from John Berdahl’s individual plant breeding nurseries at the USDA Northern Great Plains Research Laboratory in Mandan, ND. Each of these accessions represented half-sib families because seed was collected from a single plant. Evaluation of these lines continued until 1994, when at least one targeted genet from each of three selected accession plots were dug up and included in Polycross-2 with the 11 genets from Selection Nursery-1.

Polycross-2 consisted of 11 Selection Nursery-1genets that were cloned to produce 3 ramets of each individual genet and at least one targeted genet (up to a maximum of 3 potential genets due to intermingling of genets grown in a single evaluation row) from each of the three selected RI accession plots comprised of 4 genets. As was completed for Polycross-1, a segment of the evaluation row was dug from each of the 3 selected accessions from the RI Herbary. These were divided into 3 clones each and were included in Polycross-2 which was grown in the field at BFPMC. The 3 ramets/clones of each of 14 selections (11 from Selection Nursery-1 and 3 from RI accessions) were planted in a pattern to allow maximum cross pollination among all selections in the crossing block. Intermating of ramets/clones in the field occurred in1996 producing Syn-1 seeds. In fall 1997, 400 individual genets grown from Syn-1 seeds were planted at BFPMC, Big Flats, NY to form Selection Nursery-2.

#### Selection Nursery-2 and Program Ending

**Figure 1:**
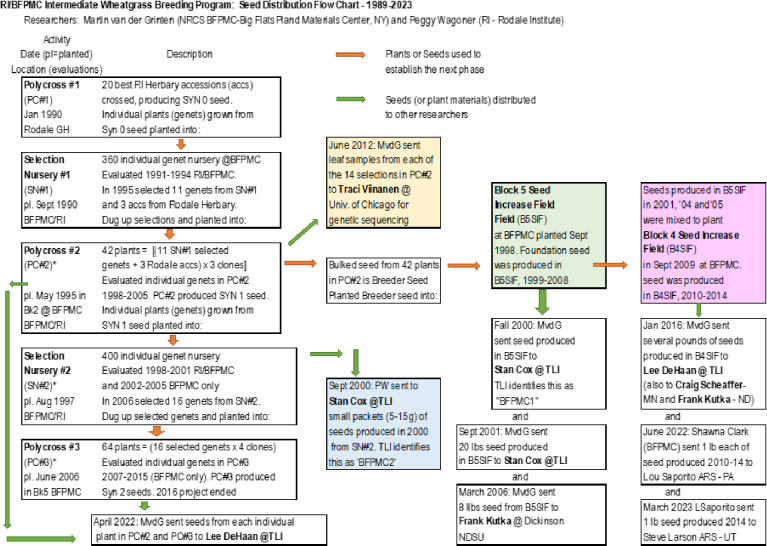
Intermediate wheatgrass germplasm flow, including germplasm distributions, from Rodale Institute Polycross-1 to Polycross-3.

Selection Nursery-2 was evaluated jointly by RI and BFPMC from 1998–2001 and only by BFPMC from 2002–2005. A total of 16 genets from Selection Nursery-2 were selected to form Polycross-3. Each genet was cloned into four ramets and planted into a crossing block in 2006. Between 2007 and 2016 BFPMC evaluated Polycross-3. Seed and materials from Polycross-2 and Selection Nursery-2 were distributed to several scientists and institutions (Fig. 1). In 2016, BFPMC exited IWG breeding.

### Genomic Analysis

#### Plant Material

To complement historical records, we leveraged existing genotype data from The Land Institute (TLI) IWG breeding program (DeHaan et al. 2018; Crain et al. 2021b, a), the NPGS IWG PI collection (Crain et al. 2023); and genotyped remnant seed from RI Polycross-2 and remnant plant material from the 14 selected Polycross-2 parents for a total of 10,341 unique genotyped genets. Within the TLI breeding program, we sampled several breeding cycles including TLI Cycle-6 (n = 3,072), TLI Cycle-12 (n = 4,032), and breeding parents from TLI Cycle 6–11 (n = 602, approximately 100 each cycle). A total of 2,329 unique genets representing 371 NPGS accessions (approximately 6 genets per accession) were genotyped previously (Crain et al. 2023), and received from NPGS web request 23159 (October 26, 2017). Known off-types and PI accessions that did not appear to morphologically resemble IWG were removed from further analysis (Crain et al. 2023). A total of 306 genets from remnant seed that was grown from Polycross-2 were genotyped. Additionally, stems, leaves, and inflorescence material in remnant seed packets of the Polycross-2 seeds were used to determine the maternal genotypes of Polycross-2 parents.

#### Genomic Profiling and Bioinformatics

DNA was extracted using a range of products including MagMAX (ThermoFisher Scientific, Waltham, MA), BioSprint (QIAGEN, Venlo, The Netherlands), and CTAB (for older tissues from Polycross-2). Across all genets, we used a two-enzyme restriction digest genotyping-by-sequencing (GBS) protocol following the methods of Poland et al. (2012). Multiplexed libraries ranging from 96 to 384 plexing were sequenced on Illumina sequencing platforms. As the samples represent a range of time and locations, sequencing platforms and output increased as technology improved. To call single nucleotide polymorphic (SNP) markers, we used the TASSEL-GBSv2 pipeline (Glaubitz et al. 2014) using the IWG genome V3.1. We filtered the marker data for strictly biallelic SNPs, a minor allele frequency greater than 0.05, and for SNPs to be called in a minimum of 30% of the genets (up to 70% missing data). Individual genets with more than 95% missing data were removed from further analysis. We required a minimum read depth of four to call a homozygote, otherwise the SNP call was set to missing if there were less than four identical reads. Heterozygous calls were allowed with a read depth of two contrasting reads. For the NPGS PI accessions where multiple genets were genotyped from each accession (Crain et al. 2023), we called SNPs on single genets as well as combining genets of a single accession to create a composite genomic profile. After filtering a total of 25,674 SNPs and 9,970 genets were used for further analysis.

We created a relationship matrix using pairwise distances calculated by the *stats* package (R Core Team 2022). From this matrix, the first and second most closely related PI accessions were determined for each improved IWG genet (TLI Cycle-6, TLI Cycle-12, breeding parents, Rodale accessions). Using 134 of the 139 RI accessions that had been genotyped, we assigned the improved IWG genets to the most likely source population (PI accession) (Manel et al. 2005). Additionally, we evaluated assignment of improved IWG genets to all genotyped (n = 370) PIs to evaluate both the strength of assignment and identify potential founders (i.e. RI accession #31) that did not have any known genotypic or historic context. To evaluate potential NPGS sources, we excluded PIs that were collected (not donated to NPGS) *ex situ* after 1990 as they would have been unavailable to RI. Because written records were quite specific that 20 accessions were used to form Polycross-1 (although accession names were not provided), we chose the 20 NPGS PI accessions that accounted for the most founder assignments of the improved IWG genets (or total number of PI accessions if less than 20) as the most likely genomic progenitors of current improved IWG germplasm for Kernza grain production. To test out ability to discriminate between relationships within and among the NPGS accessions, we used 1,997 NPGS PI genets, masked a single genet, and then assigned the genet to the most likely (closest distance) PI accession. The *ggplot2* (Wickham 2016) and *VennDiagram* (Chen 2022) R packages were used for data visualization.

## Results

### Historical Records and Experiments

Based on field and laboratory records, we documented the initial IWG breeding efforts by RI more extensively than has been previously reported by Zhang et al. (2016). Over nearly three decades RI initiated activities to develop a perennial grain crop and shared germplasm with other researchers that would eventually result in improved, food-grade IWG with products marketed under the Kernza name (Fig. 1). While the list of accessions used in Polycross-1 has been lost, these recently recovered records provide empirical data for the performance of the 20 putative accessions in Polycross-1 (Supplementary File 1). Using this information, we have identified the most likely accessions for these 20 parents (Table 1). Recovered records of the 139 accessions evaluated in 1988 and 1989 show 100 seed weight and SSR for both years but yield per 10 head data are limited to 1989 and only a few accessions from 1988. Therefore, calculated values do not perfectly align with the target value. The estimated yield per 10 heads for the most likely selected accessions is 2.67g compared to a target value of 2.5g. As there were genotype-by-environment interactions, inclusion of missing data would most likely lower the calculated value closer to the target. Based on historical records, 19 of the 20 selected accessions were from NPGS. The one other accession not from NPGS was given to RI in 1982 by Wes Jackson and TLI, but no other information is available about this accession. In terms of geographical origin, 13 of the accessions are from Russia with the majority of these collected between the Black Sea and Caspian Sea (Caspian-Pontic Steppe, Stavropol and Svetlograd regions). Passport data from NPGS suggest that three of the accessions were cultivated when collected, raising the possibility that they may all come from the same cultivar “Rostov(sky) 31” (Table 1).

### Genomic Analysis

#### Genomic Profiling of Plant Material

We combined genomic data from several different sources. Both TLI Cycle-6 and Cycle-12 were used entirely, and the breeding parents selected from TLI Cycle-6 through TLI Cycle-11 were included as they represent the direct link between TLI Cycle-6 and TLI Cycle-12. Cycle-6 from TLI represents six generations (genetic recombination and selection) between germplasm that was shared with TLI from RI (Polycross-2 and Selection Nursery 2). Likewise, another six generations beyond TLI Cycle-6 was represented by TLI Cycle-12. We used TLI Cycle-12 to both increase the number of observed assignments to NPGS PI accessions, and as this is a closed breeding program a check that PI assignments were not vastly different between the two cycles, a potential issue that would likely indicate incorrect model choice. From remnant RI material, 227 seedling tissues were evaluated along with 14 Polycross-2 maternal tissues. Finally, a total of 2,329 NPGS genets representing 370 unique PI accessions were genotyped. As genotyping was conducted on different sequencing platform and times, we evaluated the number of reads and number of SNPs called per accession. The type of data source (TLI Cycle-6, TLI Cycle-12, breeding parents, Rodale accessions, or NPGS PIs) effected the number of SNPs with an overall average of 21,028 SNPs called per accession. Single genets for the NPGS PIs had the least number of called SNPs with an average of only 10,032 SNPs followed by TLI Cycle-6 with an average of 13,883 SNPs per accession while TLI Cycle-12 have the highest average of 31,176 SNPs per accession. When the NPGS PI accessions were pooled (approximately six genets per accession), the average number of SNPs called per accession increased to 31,770. Along with number of SNPs called, we also examined the read depth of each SNP to verify that there was no apparent bias due to sequencing differences in the data sets. Cycle-6 from TLI had the lowest read depth per SNP with an average of 2.6 reads per SNP. Cycle-12 from TLI had a mean read depth of 7.6, the breeding parents were higher at 8.6, and both Rodale and NPGS PI accessions had a mean read depth of 11. When individual genets of the NPGS PIs are considered, the average read depth per genet was 1.9 (approximately 6 genets were combined per accession). These values most likely reflect older sequencing technology in TLI Cycle-6 along with different program objectives that targeted higher read depths in the breeding parents, Rodale and NPGS accessions to ensure a greater amount of data compared to the breeding program that balances practical objectives with cost.

#### Validation Test of Genet Assignments

In an effort to test the sensitivity of our assignments using the relationship matrix, we used the 1,997 genets that represented 337 unique PI IWG accessions. For each individual genet that was genotyped, we masked the known PI accession information and compared its relationship to all 337 PI accessions to identify the most related PI accession as the predicted accession. Across 1,997 iterations, only one genet was assigned to the incorrect PI accession (99% accuracy).

#### Most Likely Polycross-1 Accessions

Leveraging the genetic resources that have been developed for implementing genomics assisted breeding in IWG, we investigated the most likely founders of improved IWG from a molecular perspective. Using historical records as limits of potential germplasm, we assigned 7,786 improved IWG genets from multiple breeding cycles to their most related NPGS PI accession that was included in the initial RI evaluation. Using four different germplasm sources (Remnant Rodale, TLI Cycle-6, Breeding Parents, TLI Cycle-12), a total of 30 NPGS PI accessions were identified as the most likely founders. Of these potential matches, there was a very skewed distribution where 10 NPGS PI accessions accounted for over 98% of the assigned accessions. There were 11 NPGS PI accessions that were assigned 10 or less descendants. When considering NPGS PI assignment by germplasm source (Remnant Rodale, TLI Cycle-6, Breeding Parents, TLI Cycle-12) the most related NPGS PI accessions for the Breeding Parents and TLI Cycle-12 were a subset of NPGS PI accessions identified in TLI Cycle-6. This result should be expected as both the Breeding Parents and TLI Cycle-12 were developed from a closed population and selection would have resulted in allele frequency changes.

**Figure 2:**
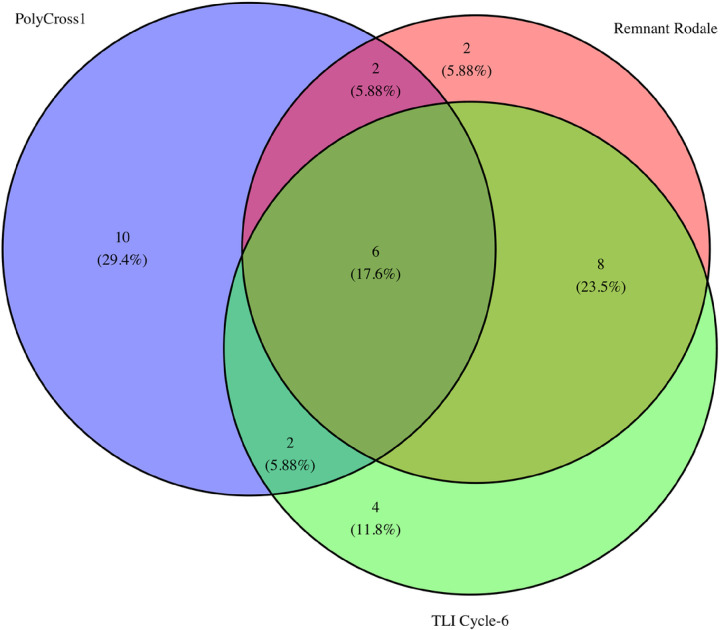
Venn diagram showing the relationship between inferred 20 intermediate wheatgrass (IWG) selections for Rodale Institute (RI) Polycross-1 (Polycross-1) and assignment based on molecular methods from two different germplasm pools. Remnant Rodale is 227 unique genets resulting from RI Polycross-2 and TLI Cycle-6 is 3,072 unique genets from The Land Institute IWG breeding program.

When comparing the most likely 20 selections in RI Polycross-1 from the recovered phenotyopic data to the inferred genets using molecular data, six NPGS PIs were identified in all data sources (NPGS PI, Remnant Rodale, TLI Cycle-6) (Fig. 2 and Tables 1 & 2). These six NPGS PIs (PI 286118, PI 273732, PI 440004, PI 314054, PI 440015, PI 316122) were the closest NPGS PI accession for over 40% of the tested genets indicating they are quite likely founders of current IWG germplasm being improved for Kernza grain production. Ten NPGS PI accessions that were identified as possible selections based on historical records for Polycross-1 did not show any descendants; however, these accessions were often part of a series of related PI accessions (i.e. PI440004–18) that were not entirely represented in the RI phenotypic analysis.

As not all the initial RI accessions (selections) were genotyped (i.e. RI Accession 31), we expanded the search of potential founders to the entire NPGS IWG collection that was collected prior to 1990. This broader search identified 51 possible NPGS sources of improved IWG germplasm. Only 10 potential NPGS accessions accounted for 93% of all assignments as the most likely founders of the tested germplasm, and just 20 NPGS PI accessions were considered the most related to more than 10 of the 7,786 tested IWG genets. Using a limit of 20 potential NPGS PI founders based on field records, a total of 39 NPGS PI accessions were identified as being the potential founders of improved IWG that is currently be used for Kernza grain production. Of these 39 accessions, just 10 accessions (Table 3) were added from the analysis considering only accessions evaluated by RI or Polycross-1 selections inferred from historical records (Tables 1 & 2).

**Figure 3:**
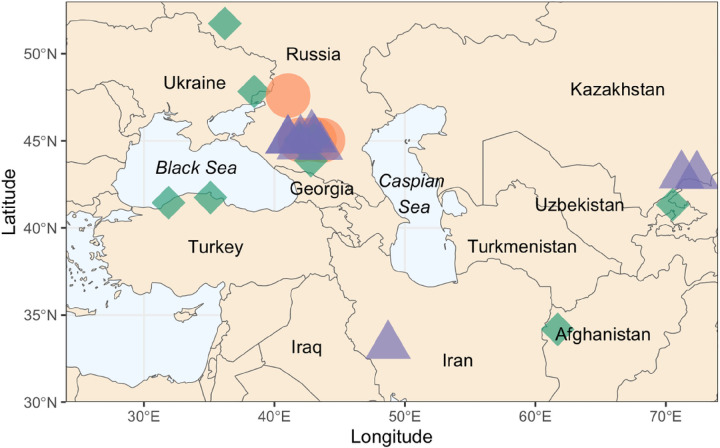
Geograpic location of 26 National Plant Germplasm System intermediate wheatgrass (IWG) plant introduction (PI) accessions that are inferred to be the founders of improved IWG germplasm used for current Kernza grain production. Orange circles represent common PIs inferred through historical and molecular methods. Green diamonds are PI accessions inferred through historic records, and purple triangles are PI inferred through molecular methods.

Finally, we investigated the most likely geographic origin of improved IWG varieties resulting in Kernza grain production using NPGS GRIN passport data. A total of 26 of 34 NPGS PI accessions (recreated RI Polycross-1 and molecular data) had reliable location data from which 19 were from Russia, two each from Turkey and Kazakhstan, and one each from Afghanistan, Iran, and Uzbekistan. Eight accessions either had missing data or location data that was deemed insufficient. For example, PI 286118 was listed with a country of origin of Denmark, yet the sample came from a botanical garden. Given this information, it is very likely this accession had been collected elsewhere before arriving in Denmark, but records of its natural origin are not available to our knowledge. The six NPGS PIs identified as possible sources of improved IWG, in all our analyses, originate from the Pontic-Caspian steppe between the Black Sea and the Caspian Sea (Fig. 3) likely indicating a primary geographic origin of improved IWG germplasm currently used for Kernza grain production.

## Discussion

### Rodale Research Activities

In the 1980s, RI initiated an effort to identify potential perennial grains and develop them into a crop. At the time this research was holistic in the treatment of what would be needed to bring a new crop to farmers. RI conducted germplasm evaluations and agronomic studies as well as partnering with outside collaborators to investigate economic, nutritional and food use analyses of IWG (Supplementary File 1). In many ways, RI developed some of the first empirical blueprints of pipeline perennial grain development that has been expanded on by later research (Wagoner 1990b; Cox et al. 2002; DeHaan et al. 2016, 2023). Additionally, the whole systems approach (breeding, agronomy, economics, end use) is similarly used today. Some examples of this integrated approach are the Kernza coordinated agriculture project (KernzaCAP, https://kernza.org/kernzacap/) and organized efforts through TLI (https://landintitute.org).

Using two years of phenotypic data RI launched a phenotypic recurrent selection program that would result in the improved germplasm used in modern Kernza grain production. Even though RI would have preferred to have had more years of evaluation for a perennial crop with an anticipated five-year crop lifetime, breeding efforts were balanced between time and institutional support. Seed shared with TLI came from Polycross-2 and Selection Nursery-2 which would have included alleles from the 20 accessions in Polycross-1 as well as genetic material from the 3 selected accessions from the Mandan breeding program (Fig. 1) (in DeHaan et al. 2018 and Bajgain et al. 2023 these are referred to as BFPMC Cycles 1 and 2 with no distinction between selection nursery and polycross seed). Much like Cox et al. suggested in 2002 that new molecular tools would aid in perennial grain development, the advent of genomic selection (Meuwissen et al. 2001) and subsequent next generation sequencing methods have allowed plant (IWG) breeding programs to harness molecular technology for improved plant breeding (Zhang et al. 2016; Bajgain et al. 2020a; Crain et al. 2021b, a). Even though RI used a minimum of two years of data to make selections, current breeding programs harnessing genomic selection can make yearly breeding selection with up to four years of phenotypic data informing the models (Crain et al. 2021b, a). Emerging technologies like speed breeding (Watson et al. 2018) have the potential to further reduce cycle time and increase the rate of genetic gain. Much like the initial RI selections that included extreme drought and wet years, genomic selection models can utilize data across multiples years and cycles (Crain et al. 2020) incorporating a range of climatic conditions into the selection information.

### Linkage Between PI Accessions and Kernza Grain Production

#### Genomic Data

Overall, the genomic data provide overwhelming support to clarify the partial historical records, which indicate that improved IWG that is currently used for Kernza grain production is primarily descended from a limited number of NPGS PI accessions mainly originating between the Black Sea and Caspian Sea. Using the genomic data, we inferred the most likely source of the 20 accessions in RI Polycross-1; however, most of the material we sampled was after Polycross-2 which had another severe bottleneck of only 14 accessions comprised of at least 14 and not more than 20 genets, Fig. 1.

Within the TLI breeding program, most of the germplasm was obtained directly from RI (Polycross-2 and Selection Nursery-2). However, there was evaluation and incorporation of several other NPGS PI accessions before TLI Cycle-6 (Bajgain et al. 2023). Within our analysis, assignment of single NPGS PI genets to their respective source NPGS PI accession was very high. Even though assignments of improved IWG genets to NPGS PI accessions appears plausible, it does not appear to be as precise as evidenced by up to 51 NPGS PI founders for improved IWG genets. Even though this number is higher than reported records, the skewed distribution suggests a number complementary with the recorded data as the source for improved IWG. Both the nature and structure of the data could be influencing these results. Perhaps the most obvious reason is that intermating and genetic recombination has blended the original NPGS accessions in such a way that it is more difficult to match the improved IWG genets to any one specific NPGS accession. As we tried to identify the most likely founders in Polycross-1, most of the material genotyped had at least two cycles of genetic recombination (remnant Rodale material) and up to 14 cycles of genetic recombination (TLI Cycle-12) from the original founders as well as the addition of genetic material from the Mandan breeding program in Polycross-2. As each recombination occurred there would have been reshuffling and breaking of the original haplotypes, and additionally there would have been selection pressure applied to obtain agronomically superior plants. Recombination and selection could alter haplotype frequencies making the analysis more challenging. Even with these potential challenges, the results bolster historical records and provide empirical evidence of a limited number of primary founders for improved IWG germplasm that is used for current Kernza grain production.

#### Potential Confounding Factors

While the historical records and genomic data had large areas of overlap, there was not complete consistency between the methods. This should not be surprising given the number of accessions evaluated, time span of the programs, and even the different institutions and staff that have been involved. In previous analysis of IWG, we have noted that more than 70% of the genetic variation observed is within accessions (Crain et al. 2023) suggesting that random sampling of seed could influence our observations. In our analysis, we have assumed that field records and that sampling was completely accurate, with similar assumptions for genetic profiling. If errors occurred, after the passage of time it would be almost impossible to identify or correct. Along with potential field errors, the IWG accessions evaluated by RI mainly came from the NPGS system after having been collected in the 1970’s and earlier. Records from NPGS indicate that there had been one seed increase since collection and distribution to RI; however, until the 1990s there was no isolation protocol in accession regenerations (Johnson et al. 1996) and IWG pollen dispersal has been documented to mainly occur within 10 m (Bajgain et al. 2022) indicating that there could have been the possibility of admixture among the NPGS accessions before they were received for genomic profiling.

Notably, both historic records and genomic data often indicate many accessions in a series. For example, the PI 4400xx series formed a large portion of the selected 20 accessions for Polycross-1. As many of the accessions were collected during the same expeditions and most likely in chronological or spatial order, it is not surprising that these accessions would often be considered more similar to each other. Work by Crain et al. (2023) showed a strong correlation between geographic distance and genetic distance within the IWG NPGS collections, suggesting that accessions collected near each other are more likely to share alleles. Even with potential germplasm, field, and laboratory errors, the data clearly indicate a small subset of IWG NPGS accessions that are the most likely primary founders of improved IWG germplasm that is currently used for Kernza grain production.

## Conclusion

This work identifies direct linkages between NPGS accessions and improved IWG germplasm that is currently grown for Kernza grain production showcasing how plant germplasm collections and repositories can be utilized for breeding and the development of new crops. By identifying the most likely genetic origins of food-grade IWG, plant breeders can continue to utilize the NPGS accessions for additional genetic diversity for enhanced crop production as well as better understand the domestication effort behind this grain. Confirming the small number of accessions within the RI breeding program that have been used to improve IWG helps indicate likely effective population size within this new crop and could guide future genetic studies. Finally, identifying the primary geographic origin as between the Black and Caspian Seas provides areas for future germplasm collections as well as *in-situ* conservation initiatives.

## Figures and Tables

**Table 1:** National Plant Germplasm System intermediate wheatgrass plant introductions (PI) inferred to be in Rodale Institute Polycross-1 from historical documents. Phenotypic evaluations are provided for seed yield trait from 1988 and 1989.

Rodale Accession	Accession Source	Plant introduction NPGS data		Rodale Institute Phenotypic Evaluation					
		Origin, Collection site	Status^†^	100 Seed weight (g)		Seed Set Rating %		Yield (g) per 10 heads	
(elevation (m))	1988	1989	1988	1989	1988	1989			
867	**PI 273732**	Russia, Rostov	C ‘Rostovsky 31’	0.41	0.46	43	46		2.81
869	**PI 286118**	Denmark	U	0.49	0.52	45	49		3.15
873	**PI 314054**	Russia, Rostov	U	0.42	0.52	42	50		3.64
875	**PI 315353**	Russia, Voronezh region	U	0.42	0.42	51	38		2.92
877	**PI 315355**	Russia, Stavropol region	W	0.50	0.48	39	51		3.36
878	**PI 316122**	Former USSR	U	0.51	0.55	49	46	2.89	2.99
833	PI 326209	Former USSR Atai region	W	0.55	0.44	51	43	3.95	2.02
785	PI 401201	Iran, SE of Khorranabad (1600)	W	0.46	0.55	45	52		2.85
793	**PI 440004**	Russia	C ‘Rostov 31’	0.51	0.49	48	47	3.03	2.79
797	PI 440008	Russia, SE of Stavropol (400)	W	0.42	0.50	44	47		2.75
800	**PI 440011**	Russia, near Svetlograd (250)	W	0.57	0.45	44	47		2.80
898	PI 440014	Russia, SE of Svetlograd	W	0.52	0.45	46	45	2.14	1.89
899	**PI 440015**	Russia, SE of Svetlograd (300)	C likely ‘Rostov 31’	0.55	0.49	50	47	3.46	2.60
901	**PI 440017**	Russia, SE of Svetlograd (300)	W	0.52	0.52	49	41	2.49	2.10
836	PI 440028	Russia, SE of Stavropol (500)		0.50	0.43	43	35		1.25
837	PI 440029	Russia, E of Stavropol (600)	W	0.53	0.40	43	31		1.25
839	PI 440031	Russia, E of Stavropol (400)	W	0.59	0.50	45	37	2.89	1.62
918	PI 440038	Kazakhstan, SE of Dzhambul (1080)	W	0.57	0.40	51	36	3.67	1.98
846	PI 440039	Kazakhstan, SE of Dzhambul (900)	W	0.64	0.56	45	31	2.51	1.21
31	Wes Jackson			0.53	0.51	51	48	2.02	2.54

† Wild (W), Uncertain (U), Cultivated with variety name (C)

PIs in bold were also identified using molecular methods as most likely included in Polycross-1.

**Target values** and actual overall (1988 and 1989) *average* of 20 accessions: **0.495** *0.496* **45** *44.8* **2.50** *2.67*

**Table 2:** National Plant Germplasm System intermediate wheatgrass plant introductions (PI) inferred to be in Rodale Institute Polycross-1 from molecular data. Phenotypic evaluations are provided for seed yield trait from 1988 and 1989.

Rodale Accession	Accession Source	Plant introduction NPGS data		Rodale Accession				
		Origin, Collection site	Status^†^	100 Seed weight (g)		Seed Set Rating %		Yield (g) per 10 heads
(elevation (m))	1988	1989	1988	1989	1988	1989		
863	PI210992	Afghanistan, Herat Province	W	0.62	0.46	38	37	1.15
868	PI273733	Russia, Kursk Oblast	U	0.37	0.43	42	49	2.01
822	PI297876	Russia	U	0.5	0.42	40	22	0.75
827	PI314192	Uzbekistan, E Tashkent	W	0.55	0.43	39	35	1.36
830	PI315065	Former Soviet Union	U	0.47	0.41	40	38	2.14
874	PI315067	Former Soviet Union	U	0.42	0.34	41	42	3.01
876	PI315354	Russia, Stavropol	W	0.47	0.48	46	42	2.06
831	PI325190	Russia, Stavropol	W	0.47	0.29	38	19	0.71
882	PI345586	Russia, Rostov Region	C‘Rostovskii 31’	0.43	0.42	47	53	2.52
887	PI401014	Turkey, Zongulkad (60)	W	0.5	0.36	42	40	1.35
755	PI401015	Turkey, Sinop (30)	W	0.39	0.24	41	10	0.2
794	PI440005	Russia, Stavropol	C‘Stavropol-10’	0.4	0.42	38	43	2.56
798	PI440009	Russia, E Stavropol (600)	W	0.42	0.46	39	37	1.44
902	PI440018	Russia, SE Svetlograd (300)	W	0.44	0.44	46	41	2.28

† Wild (W), Uncertain (U), Cultivated with variety name (C)

**Target values** and actual overall (1988 and 1989) *average* of these 14 accessions: **0.495** *0.301***45** 27.1 **2.50** *1.18*

**Table 3:** National Plant Germplasm System intermediate wheatgrass (IWG) plant introductions that were not evaluated by Rodale Institute but share similarity with improved IWG germplasm for Kernza grain production in current breeding programs.

Accession Source	Plant introduction NPGS data	
Origin, Collection site	Status^†^	
(elevation (m))		
PI173630	Turkey, Solhan	W
PI486197	Turkmenistan	W
PI502351	Russia, Elista (120)	C
PI502356	Russia, Stvropol (500)	C
PI547315	Russia, Leningrad Oblast	W
PI547318	Russia	C
PI547319	Russia, Leningrad Oblast	W
PI547334	Poland	W
PI574518	USA	Breeding Material
PI578695	USA	Breeding Material

† Wild (W), Cultivated with variety name (C)

**Supplementary File 1**

Wagoner, P., Crain, J., Larson, S., & DeHaan, L. Origin of current intermediate wheatgrass germplasm being developed for Kernza grain production. Submitted to Genetic Resources and Crop Evolution.

Primary sources evaluated to reconstruct the Rodale Institute efforts to develop a perennial grain. While some have been cited in the main text, all sources have been kept along with classification as to the type of information each source contains. Note: the classification is broadscale and additional information on other research areas may be included in each source.

Classified as:

a. Species evaluated by Rodale Institute

b. Potential food utilization of intermediate wheatgrass

c. Rodale research toward perennial grain development (experiment and research methodology)

d. Intermediate wheatgrass germplasm evaluation

1. Becker, R., G.D. Hanners, D. W. Irving and R.M. Saunders. 1986. Chemical composition and nutritional qualities of five perennial grains. Food Sci. and Techn. 19:312–315. **(b)**

2. Becker, R., P. Wagoner, G.D. Hanners, R.M. Saunders. 1991. Compositional, nutritional and functional evaluation of intermediate wheatgrass (*Thinopyrum intermedium).* J Food Proc. and Preserv. 15:63–77. **(b)**

3. Becker, R., P. Wagoner, T.J. Bargman. J.H. Rupnow and R.M. Saunders. 1990. New food grains for sustainable agricultural systems. In Proceedings, Sustainable Agriculture in California: A Research Symposium, University of California, Davis, CA. **(b)**

4. Hanners, G.D., Becker, R., Irving, D.W. and Saunders, R.M. 1988. Nutritional qualities of perennial grains with agricultural potential. in Global Perspectives on Agroecology and Sustainable Agricultural Systems, Proceedings of the Sixth International Scientific Conference of the International Federation of Organic Agricultural Movements (IFOAM). Vol. 2, P. Allen and D. Van Dusen, Eds., Agroecology Program, University of California, Santa Cruz, CA. **(b)**

5. Harrington, M. and P. Wagoner. 1986. Food applications for perennial grains. Poster presented at the 1986 Annual meeting of the American Association of Cereal Chemists, Toronto, Ontario, Canada. **(b)**

6. Irving, D.W., J.L. Peake, V.A. Breda. 1990. Nutrient distribution in five perennial grain species exhibited by light and scanning electron microscopy. Cereal Chem. 68(4):376–382. **(b)**

7. Marshall, S.W. 1971. An evaluation of maternal line selection and mass selection with pollen control in Russian Wildrye. Ph.D. Dissertation, University of Idaho. Moscow, ID. 63 pp. **(c)**

8. North Dakota State University/Rodale Research Center. 1989. Grass or Grain? Intermediate Wheatgrass in a Perennial Cropping System for the Northern Great Plains. P. Wagoner, J.C. Gardner, B.G. Schatz, F. Sobolik and D. Watt, contributors, North Dakota Agricultural Experiment Station Research Report No. 108. Fargo, ND **(c)**

9. Schauer, A. 1989. Evaluation of Intermediate Wheatgrass Accessions: 1988 Summary. RRC/NC89/34. Rodale Press, Inc. Emmaus, PA. **(d)**

10. Schauer, A. 1990. Evaluation of Intermediate Wheatgrass Germplasm: 1989 Summary. RRC/NC90/34. Rodale Press, Inc. Emmaus, PA. **(c, d, contains most of the 1988 and 1989 evaluation data used for Polycross-1)**

11. Schauer, A. and P. Wagoner. 1986 Perennial Grain Research: Large Scale Simulation Trials. 1984 and 1985. RRC/NC-86/32. Rodale Press, Inc. Emmaus, PA. **(c)**

12. Slinkard, A.E. 1965. Fertility in intermediate wheatgrass, *Agropyron intermedium* (Host)Beauv. Crop Science. 5:363–365. **(c)**

13. The Morning Call, Dec 29, 1988. Drought, heat, killing of CAT fund topped PA stories '88 the year in review. Allentown, PA. **(c)**

14. Trupp, C.R., 1965. A diallel analysis of seed yield components in intermediate wheatgrass, *Agropyron intermedium* (Host) Beauv. Unpublished M.S. Thesis, University of Idaho, Moscow, ID. 124 pp. **(c)**

15. Trupp, C.R. and A.E. Slinkard. 1965. Seedset rating as a measure of fertility in grasses. Crop Science. 5:599–600. **(c)**

16. Wagoner, P. 1984. Summary of the Genera Observed in the 1983 Perennial Herbary. RRC/NC- 84/30. Rodale Press, Inc., Emmaus, PA. **(a, c)**

17. Wagoner, P. 1986. The potential for grain production from perennial forage grasses. In Proceedings of the Twenty-ninth Grass Breeders Work Planning Conference. Northern Great Plains Research Center, USDA-aRs, Mandan, ND. **(a)**

18. Wagoner, P. 1987. Summary of Research: Development of Perennial Grain Cropping Systems at the Rodale Research Center (1983–1986). RRC/NC-87/33. Rodale Press, Inc., Emmaus, PA. **(a)**

19. Wagoner, P. 1988. Perennial Grain Research at the Rodale Research Center: 1987 Summary. RRC/nC-88/33. Rodale Press, Inc. Emmaus, PA. **(b, d)**

20. Wagoner, P. 1988. Perennial grain research at the Rodale Research Center. In Global Perspectives on Agroecology and Sustainable Agricultural Systems, Proceedings of the Sixth International Scientific Conference of the International Federation of Organic Agricultural Movements (IFOAM). Vol. 2, P. Allen and D. Van Dusen, Eds., Agroecology Program, University of California, Santa Cruz, CA. **(c)**

21. Wagoner, P. 1989. The Study of Intermediate Wheatgrass as a Perennial Grain Crop: 1988 Summary. rRc/NC-89/33. Rodale Press, Inc., Emmaus, PA. **(c, d, includes some 1988 evaluation data used for Polycross-1)**

22. Wagoner, P. 1990. Perennial Grain Development: Past Efforts and Potential for the Future. Critical Reviews in Plant Science. 9(5):381–409. **(c)**

23. Wagoner, P. 1990. Perennial Grain new use for intermediate wheatgrass. J. Soil and Water Conserv. 45(1):81–82. **(c)**

24. Wagoner, P. 1991. Evaluation of Intermediate Wheatgrass Germplasm: 1990 Summary. RIRC/AG91/34. Rodale Institute, Kutztown, PA. **(d)**

25. Wagoner, P. 1995. Intermediate wheatgrass (*Thinopyrum intermedium)* development of a perennial grain crop. in Cereals and Psuedocereals. J.T. Williams, Ed. Chapman and Hall, New York, NY. **(c)**

26. Wagoner, P. and A.L. Schauer. 1990. Intermediate wheatgrass as a perennial grain crop. in Advances in New Crops. J. Janick and J. Simon, Eds., Timber Press, Portland, OR. **(c)**

27. Wagoner, P. and A. Schauer. 1987. Large Scale Perennial Grain Production Simulation Plots: 1986 Summary. rRc/NC-87/32. Rodale Press, Inc. Emmaus, PA. **(c)**

28. Wagoner, P. and A. Schauer. 1988. Perennial Grain Production Trials at the Rodale Research Center: 1987 Summary. RRC/NC-88/32. Rodale Press, Inc. Emmaus, PA. **(c)**

29. Wagoner, P. and A. Schauer. 1989. Intermediate Wheatgrass Grain Production Trials at the Rodale Research Center: 1988 Summary. RRC/NC-89/32. Rodale Press, Inc. Emmaus, PA. **(c)**

30. Wagoner, P., A.L. Schauer, L Teerlinck. 1992. Intermediate wheatgrass (*Thinopyrum intermedium):* a potential perennial grain. Poster presented at International Conference on Development of New Crops, Jerusalem, Israel. **(c)**

31. Wagoner, P., A.L. Schauer and R.R. Janke. 1990. Perennial grain development: the potential of intermediate wheatgrass. Poster presented at the 1990 Annual meeting of American Society of Agronomy, Crop Science Association, Soil Science Society of America, San Antonio, TX. **(c)**

32. Wagoner, P., M. van der Grinten and L.E. Drinkwater. 1996. Breeding intermediate wheatgrass (Thinopyrum intermedium) for use as a perennial grain. Poster presented at the 1996 Annual meeting of American Society of Agronomy, Crop Science Association, Soil Science Society of America, Indianapolis, IN. **(c, d, includes the average values for the 20 selected accession in Polycross-1)**

33. Wagoner, P., R. Becker, A.P. Mossman and R.M. Saunders. 1989. Perennial wheat relative as a new food grain. Poster presented at the 1989 Annual meeting of the American Association of Cereal Chemists, Washington, D.C. **(b)**

34. Wood, M. 1991. The Great Wheatgrass Bake-Off. Agricultural Research. USDA-ARS. July 1991:18–19. **(b)**

## Data Availability

DNA sequence data generated for this study was uploaded to the NCBI Sequence Read Archive (SRA) (https://www.ncbi.nlm.nih.gov/bioproect) as bioproject. Data from bioproject PRJNA609325were also used for analysis. All data scripts and data files have been placed in the Zenodo digital repository doi. (For peer review, materials can be accessed at http://people.beocat.ksu.edu/~jcrain/Kernza_Origin)

## Abbreviations

ARS: Agriculture Research Service
BFPMC: Big Flats Plant Materials Center, United States Department of Agriculture Natural Resources Conservation Service, Big Flats, NY
GBS: genotyping-by-sequencing
GS: genomic selection
IWG: intermediate wheatgrass
NPGS: National Plant Germplasm System
PI: plant introduction
RI: Rodale Institute
SNP: single nucleotide polymorphism
SSR: seed set rating
TLI: The Land Institute
